# Muscle ultrasound and shear wave elastography for screening sarcopenia in patients undergoing maintenance hemodialysis: a prospective study

**DOI:** 10.3389/fmed.2026.1871173

**Published:** 2026-08-05

**Authors:** Jinxiao Chen, Yaqin Feng, Yamie Xie, Lixia You, Linyan Ke

**Affiliations:** 1 Department of Ultrasound, Lishui Hospital of Wenzhou Medical University, The First Affiliated Hospital of Lishui University, Lishui People’s Hospital, Lishui, Zhejiang, China; 2 Department of Radiology, Jinjiang Municipal Hospital, Jinjiang City, Fujian, China; 3 Zhejiang Chinese Medical University, Hangzhou, Zhejiang, China; 4 Department of Nephrology, Lishui Hospital of Wenzhou Medical University, The First Affiliated Hospital of Lishui University, Lishui People’s Hospital, Lishui, Zhejiang, China

**Keywords:** elasticity imaging techniques, nutritional status, renal dialysis, sarcopenia, ultrasonography

## Abstract

**Background:**

Sarcopenia is highly prevalent among patients undergoing maintenance hemodialysis (MHD) and is associated with adverse clinical outcomes. However, practical and reliable screening tools remain limited. This study aimed to evaluate the feasibility and diagnostic value of musculoskeletal ultrasound for sarcopenia assessment in MHD patients.

**Methods:**

In this prospective single-center study, 126 patients undergoing chronic hemodialysis were classified into sarcopenia and non-sarcopenia groups according to established diagnostic criteria. Musculoskeletal ultrasound and shear wave elastography (SWE) were performed to assess morphological and mechanical properties of multiple muscle groups, including the rectus femoris, vastus intermedius, rectus abdominis, medial gastrocnemius, and biceps brachii. Measurement repeatability was evaluated using intraclass correlation coefficients (ICCs). Independent predictors of sarcopenia were identified using multivariable logistic regression, and diagnostic performance was assessed using receiver operating characteristic (ROC) analysis.

**Results:**

Ultrasound measurements demonstrated excellent reproducibility, with ICCs ranging from 0.869 to 0.995. Compared with non-sarcopenic patients, those with sarcopenia exhibited significant alterations in several ultrasound-derived muscle parameters, particularly rectus femoris cross-sectional area (RF-CSA), rectus femoris shear wave velocity (RF-SWV), and biceps brachii measurements. In the conventional ultrasound model, sex, hemodialysis duration, RF-CSA, BB-SFT, and BB-CSA were identified as independent predictors of sarcopenia, achieving an area under the curve (AUC) of 0.944. After incorporating SWE parameters, duration of hemodialysis, RF-CSA, and RF-SWV remained significant predictors, and combined model demonstrated improved discriminative performance (AUC = 0.994).

**Conclusion:**

Musculoskeletal ultrasound demonstrated good reproducibility and promising diagnostic performance for identifying sarcopenia in patients undergoing maintenance hemodialysis. The addition of shear wave elastography may further improve screening performance. These findings suggest that ultrasound-based assessment may serve as a practical and noninvasive screening approach for sarcopenia in this population, although further validation in larger multicenter cohorts is warranted.

## Introduction

Chronic kidney disease (CKD) represents a major global health burden, and patients receiving maintenance hemodialysis (MHD) are particularly vulnerable to a wide range of systemic complications that adversely affect survival and quality of life (1–4). Among these, protein-energy wasting, chronic inflammation, metabolic disturbances, and reduced physical activity contribute to profound alterations in body composition, particularly the progressive loss of skeletal muscle mass and function (5–8). These changes are closely linked to sarcopenia, a multifactorial skeletal muscle disorder characterized by declines in muscle quantity, strength, and performance, which has emerged as a critical determinant of adverse outcomes in the dialysis population (9–12).

The prevalence of sarcopenia in patients undergoing MHD is substantially higher than that observed in the general population, with reported rates ranging from approximately 25% to over 30% depending on diagnostic criteria and study populations (13, 14). Accumulating evidence indicates that sarcopenia in MHD patients is associated with increased risks of frailty, hospitalization, cardiovascular events, and mortality (14–16). Therefore, early identification and accurate assessment of sarcopenia are essential for risk stratification and the implementation of targeted interventions in this high-risk group (17, 18).

Despite growing recognition of its clinical importance, the assessment of sarcopenia in MHD patients remains challenging. Current consensus guidelines, including those from the Asian Working Group for Sarcopenia (AWGS) and the European Working Group on Sarcopenia in Older People (EWGSOP2), recommend a multidimensional diagnostic framework incorporating muscle mass, muscle strength, and physical performance (19–21). However, the clinical application of these criteria is often limited by the availability and practicality of conventional measurement techniques (22). Dual-energy X-ray absorptiometry (DXA), commonly used to quantify muscle mass, may be influenced by fluid overload in dialysis patients, potentially leading to measurement bias. Bioelectrical impedance analysis (BIA), although convenient for bedside use, provides indirect estimates of muscle mass and is similarly affected by hydration status. Computed tomography (CT) and magnetic resonance imaging (MRI), while considered reference standards, are costly, time-consuming, and not routinely feasible in clinical practice (20).

In recent years, musculoskeletal ultrasound has emerged as a promising modality for the evaluation of skeletal muscle (23). Owing to its noninvasive nature, portability, real-time imaging capability, and cost-effectiveness, ultrasound offers a practical alternative for bedside assessment in dialysis settings. Conventional two-dimensional ultrasound (2D-US) parameters, such as muscle thickness (MT) and cross-sectional area (CSA), have been widely applied to estimate muscle quantity and have demonstrated good correlation with established imaging techniques (24–26). In CKD and MHD patients, most ultrasound based studies focus on a single muscle. However, muscle atrophy in dialysis patients is a systematic process, and a comprehensive evaluation of multiple muscle groups may more accurately reflect the overall state (27–29).

Beyond structural assessment, shear wave elastography (SWE) technology can quantitatively evaluate tissue stiffness by measuring the shear wave velocity (SWV) of the tissue (30, 31). This provides additional insights into the mechanical properties of muscles, including fibrosis and changes in muscle composition, which cannot be captured by traditional morphological parameters.

Preliminary research suggests that SWE may improve the characterization of subtle muscle alterations and enhance the diagnostic performance of ultrasound-based muscle assessment (32–35). However, there is still limited research on its application in MHD patients with sarcopenia.

Therefore, the present study aimed to comprehensively evaluate both conventional 2D ultrasound and SWE parameters across multiple skeletal muscle groups, including the rectus femoris, vastus intermedius, rectus abdominis, gastrocnemius, and biceps brachii, in patients undergoing chronic hemodialysis. Furthermore, this study sought to investigate the association between ultrasound-derived muscle parameters and sarcopenia status, compare imaging characteristics between sarcopenic and non-sarcopenic individuals, and determine the diagnostic value of musculoskeletal ultrasound as a practical screening tool for sarcopenia in the MHD population.

## Materials and methods

### Study participants and design

This prospective cross-sectional study was conducted at Lishui People’s Hospital, China, from January to April 2025. The study protocol was reviewed and approved by the Ethics Review Committee of Lishui People’s Hospital (Approval No. 2025-083-01). The study was conducted in accordance with the principles of the Declaration of Helsinki. Written informed consent was obtained from all participants prior to enrollment.

**FIGURE 1:**
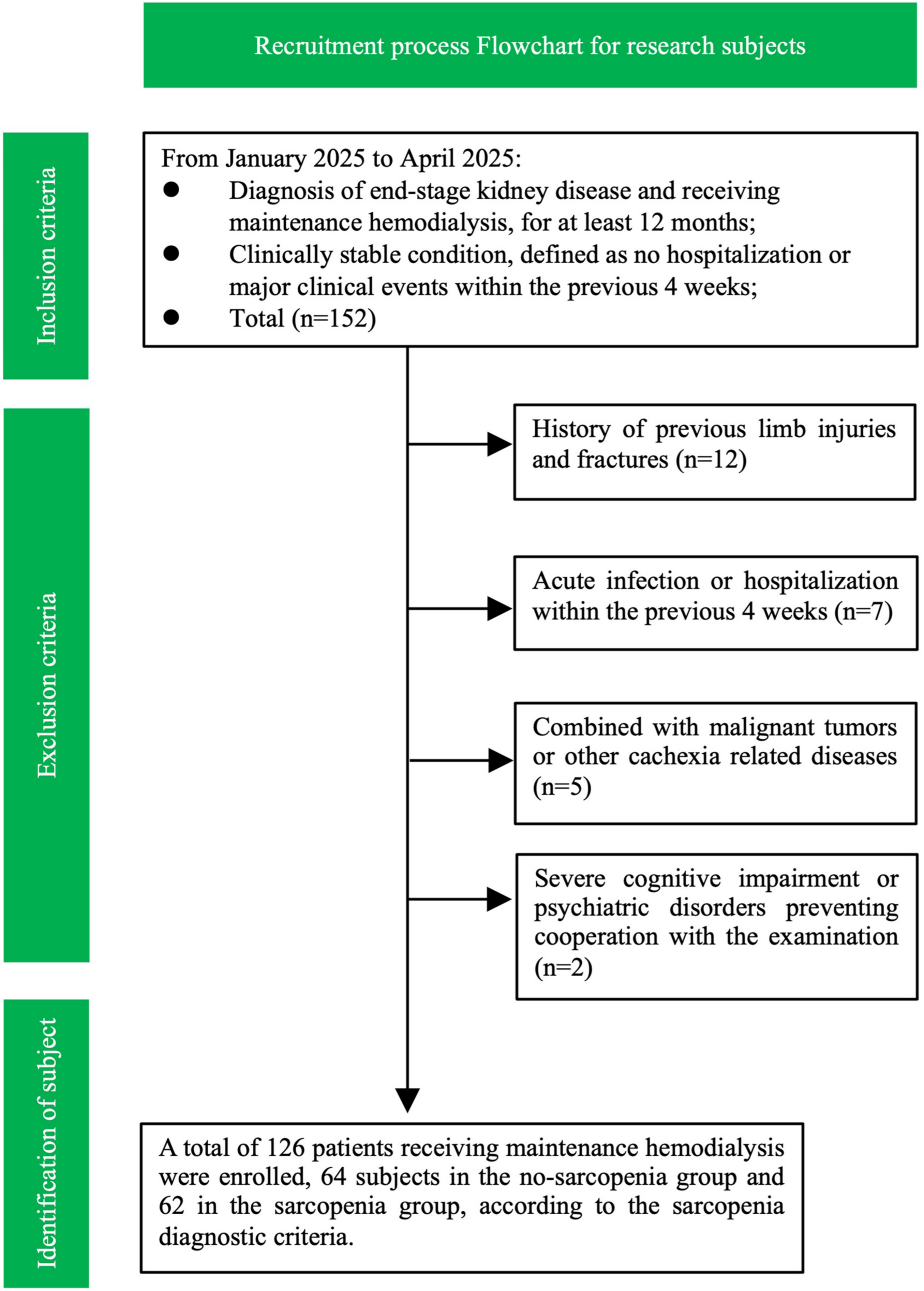
Subject recruiting flowchart.

The inclusion and exclusion criteria are detailed in the flowchart (Figure 1). All participants underwent trunk muscle ultrasound examination to evaluate muscle structure, function, and elastic properties. To ensure the reliability of ultrasound results, muscle ultrasound repeatability evaluation was performed on 30 subjects.

This study was reported in accordance with the Strengthening the Reporting of Observational Studies in Epidemiology (STROBE) guidelines.

### Study sample size estimation

Sample size estimation was performed according to the events-per-variable (EPV) principle commonly used in multivariable logistic regression analyses to minimize overfitting and ensure model stability. Assuming an expected event rate of 0.5 and accounting for a 10% dropout rate, a minimum sample size of 120 participants was estimated.

### Recruitment criteria for study participants

All participants were recruited voluntarily at Lishui People’s Hospital. In the study evaluating ultrasound screening for sarcopenia in chronic hemodialysis patients, a total of 126 chronic hemodialysis patients were recruited, and reproducibility assessment was conducted in 30 of them. The recruitment process of the subjects is shown in Figure 1.

Participants were enrolled if they met all of the following criteria: (1) age ≥ 18 years; (2) diagnosis of end-stage kidney disease (ESKD) and receiving maintenance hemodialysis, for at least 12 months; (3) clinically stable condition, defined as no hospitalization or major clinical events within the previous 4 weeks; (4) ability to maintain a supine or sitting position and cooperate with ultrasound examination; and (5) availability of complete clinical and laboratory data.

Participants were excluded if they met any of the following criteria: (1) presence of neuromuscular disorders or conditions known to significantly affect muscle mass or function (e.g., myopathy, motor neuron disease, myasthenia gravis, or stroke with residual motor dysfunction); (2) severe musculoskeletal disorders affecting the limbs (e.g., fracture, deformity, or amputation); (3) acute infection, systemic inflammatory condition, or hospitalization within the previous 4 weeks; (4) recent surgery or major trauma within the previous 3 months; (5) active malignancy or other cachexia-associated diseases; (6) local conditions interfering with ultrasound measurements, including severe subcutaneous edema, skin infection, or wounds at the measurement site; (7) severe cognitive impairment or psychiatric disorders preventing cooperation with the examination; and (8) incomplete or missing key clinical, laboratory, or ultrasound data.

### Laboratory measurements

Fasting venous blood samples were collected from all participants prior to dialysis sessions and analyzed at the central laboratory of Lishui People’s Hospital using standardized automated methods with established internal quality control procedures.

The following biochemical parameters were recorded: blood urea nitrogen (BUN), serum creatinine (Cr), albumin, hemoglobin (Hb), parathyroid hormone (PTH), serum phosphorus, uric acid (UA), total cholesterol (TC), high-density lipoprotein cholesterol (HDL-C), and low-density lipoprotein cholesterol (LDL-C).

These laboratory variables were included to comprehensively characterize patients’ renal function, nutritional status, and metabolic profile. They were used for baseline comparisons between groups and correlation analyses, and were initially considered as candidate variables in univariate analyses. Only variables meeting predefined statistical selection criteria were subsequently included in the final multivariable logistic regression model, in order to reduce overfitting and ensure model parsimony.

### Ultrasound assessment of muscles

**FIGURE 2:**
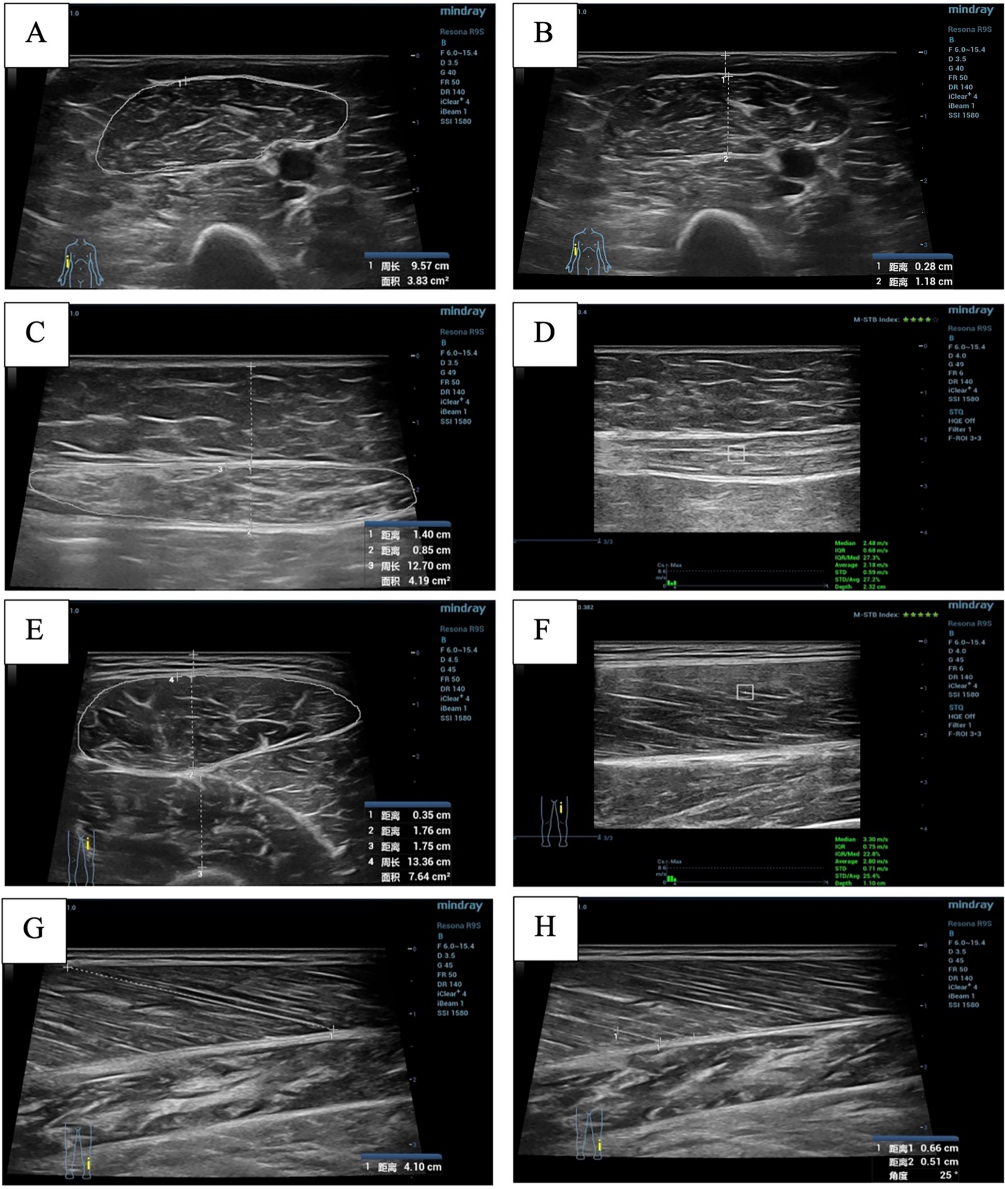
Representative ultrasound measurements of skeletal muscle parameters. **(A)** Measurement of biceps brachii cross-sectional area (BB-CSA). **(B)** Measurement of biceps brachii muscle thickness (BB-MT) and subcutaneous fat thickness (BB-SFT). **(C)** Measurement of rectus abdominis muscle thickness (RA-MT), cross-sectional area (RA-CSA), and subcutaneous fat thickness (RA-SFT). **(D)** Measurement of rectus abdominis shear wave velocity (RA-SWV). **(E)** Measurement of rectus femoris muscle thickness (RF-MT), cross-sectional area (RF-CSA), subcutaneous fat thickness (RF-SFT), and vastus intermedius muscle thickness (VI-MT). **(F)** Measurement of rectus femoris shear wave velocity (RF-SWV). **(G)** Measurement of medial gastrocnemius fascicle length (MG-ML). **(H)** Measurement of pennation angle (PA) of the medial gastrocnemius.

Ultrasound examinations were performed in dialysis wards and ultrasound clinics using a Resona R9T ultrasound system (Mindray Biomedical Electronics, Shenzhen, China) equipped with a high-frequency linear-array transducer (L14-3WU, 5–18 MHz). All two-dimensional shear wave elastography (2D-SWE) measurements were obtained using the same system, as shown in Figure 2.

To minimize the potential influence of fluid overload on muscle morphology and elasticity measurements, all ultrasound examinations were performed within 1–2 h after completion of the routine hemodialysis session, when patients had achieved their clinically prescribed dry weight. This standardized timing was selected to reduce variability related to hydration status and improve the reliability of ultrasound assessments.

All examinations were conducted by two experienced sonographers (each with > 5 years of experience in musculoskeletal ultrasound). Prior to study initiation, both sonographers underwent standardized training and followed the same predefined scanning protocol and measurement procedures throughout the study to ensure consistency across examinations. Participants were instructed to remain relaxed throughout the examination, and excessive transducer pressure was avoided to minimize tissue compression.

#### Ultrasound examination method for rectus abdominis muscle

For the assessment of the rectus abdominis, participants were positioned supine with relaxed abdominal muscles. The probe was placed transversely, slightly to the right of the umbilicus, and oriented perpendicular to the muscle fibers to obtain a cross-sectional image. On this plane, rectus abdominis muscle thickness (RA-MT), subcutaneous fat thickness (RA-SFT), and cross-sectional area (RA-CSA) were measured.

Subsequently, a 3 × 3 mm region of interest (ROI) was placed at the center of the muscle belly at a depth of approximately 1–3 cm for SWE measurement. Each measurement was repeated three to five times, and the mean value was recorded as rectus abdominis shear wave velocity (RA-SWV).

#### Ultrasound examination methods for rectus femoris and rectus femoris muscles

For the evaluation of the rectus femoris and vastus intermedius, participants remained in the supine position with the lower limbs extended and relaxed. The probe was placed at the midpoint between the anterior superior iliac spine and the superior border of the patella, perpendicular to the muscle fibers, to obtain a transverse image. Rectus femoris muscle thickness (RF-MT), subcutaneous fat thickness (RF-SFT), and cross-sectional area (RF-CSA) were measured on this plane. The vastus intermedius, located between the rectus femoris and the femur, was identified on the same image, and its muscle thickness (VI-MT) was measured.

SWE was then performed by placing a 3 × 3 mm ROI at the center of the rectus femoris muscle belly at a depth of 1–3 cm. Measurements were repeated three to five times, and the mean value was recorded as rectus femoris shear wave velocity (RF-SWV).

Due to the deep anatomical location of VIM, the depth can affect the size of SWV values. To ensure comparability of SWV values between muscles, this study did not measure the SWV values of VIM.

#### Ultrasound examination methods for medial head of gastrocnemius muscles

All measurements were conducted with patients in a prone position with both feet naturally suspended at the edge of the examination table to ensure that the muscles are in a relaxed state. The measurement site is 30% of the distance from the popliteal fold to the lateral ankle, and longitudinal ultrasound images are obtained to ensure clear visualization of the muscle bundle and aponeurosis.

Medial gastrocnemius muscle thickness (MG-MT) was defined as the vertical distance between the superficial and deep aponeuroses. Medial gastrocnemius fascicle length (MG-ML) was defined as the length of a visible muscle fascicle between the superficial and deep aponeuroses, with linear extrapolation applied when the entire fascicle was not fully visualized. Subcutaneous fat thickness (MG-SFT) was defined as the distance between the skin surface and the superficial fascia. Pennation angle (PA) was defined as the angle between the muscle fascicle and the deep aponeurosis.

Next, similarly, SWE measurements were obtained by placing a 3 × 3 mm ROI at the center of the muscle belly at a depth of 1–3 cm. Each measurement was repeated three to five times, and the mean value was recorded as medial gastrocnemius shear wave velocity (MG-SWV).

These parameters were measured on longitudinal ultrasound images to ensure clear visualization of the fascia while avoiding excessive probe pressure on the subcutaneous tissue.

#### Ultrasound examination methods for biceps brachii muscle

For the biceps brachii, participants were positioned supine with the upper limbs relaxed and the palms facing upward. The ultrasound probe was placed at the midpoint of the upper arm, perpendicular to the muscle fibers, to obtain a cross-sectional image. Biceps brachii muscle thickness (BB-MT), cross-sectional area (BB-CSA), and subcutaneous fat thickness (BB-SFT) were measured on this plane.

SWE was subsequently performed by placing a 3 × 3 mm ROI at the center of the muscle belly at a depth of 1–3 cm. Each measurement was repeated three to five times, and the mean value was recorded as biceps brachii shear wave velocity (BB-SWV).

### Inter-observer repeatability assessment

To evaluate inter-observer reproducibility, 30 subjects were randomly selected. Two experienced sonographers independently performed ultrasound examinations using the same ultrasound system according to the standardized scanning protocol. The interval between the two examinations did not exceed 30 min, and both operators were blinded to each other’s measurement results. After completing the examinations of all subjects, the ultrasound results of the two groups were organized and the inter-observer agreement was evaluated using the intra-class correlation coefficient (ICC).

### Diagnosis of sarcopenia in MHD patients

Sarcopenia was diagnosed primarily according to the criteria proposed by the Asian Working Group for Sarcopenia (AWGS 2019), which were selected because they are specifically developed and validated for Asian populations. The European Working Group on Sarcopenia in Older People (EWGSOP2) recommendations were cited as a complementary conceptual framework supporting the multidimensional assessment of muscle strength, muscle mass, and physical performance (19, 20). In the present study, all diagnostic cutoff values, including handgrip strength, appendicular skeletal muscle index (ASMI), and physical performance measures, were based exclusively on the AWGS 2019 criteria.

#### Assessment of muscle strength

Muscle strength was evaluated using handgrip strength, measured with an electronic dynamometer (EH101, SENSSUN Group Limited, Guangzhou, China). Participants were seated with the elbow flexed at approximately 90°, and measurements were obtained from the dominant hand. Three consecutive trials were performed, and the highest value was recorded for analysis.

According to AWGS 2019 criteria, low muscle strength was defined as a handgrip strength of < 28 kg in men and < 18 kg in women.

#### Assessment of muscle mass

Muscle mass was assessed using dual-energy X-ray absorptiometry (DXA) based on the appendicular skeletal muscle index (ASMI), as recommended by the Asian Working Group for Sarcopenia. This approach focuses on skeletal muscle mass of the extremities rather than whole-body composition, providing a practical and validated indicator of muscle quantity.

Appendicular lean mass (ALM) was obtained from limb measurements and normalized to height squared (kg/m^2^) to calculate ASMI. According to the AWGS 2019 criteria, low muscle mass was defined as an ASMI of < 7.0 kg/m^2^ in men and < 5.4 kg/m^2^ in women.

#### Assessment of physical performance

Physical performance was evaluated using either the 4-m gait speed test or the Short Physical Performance Battery (SPPB), depending on patient condition.

For gait speed assessment, participants were instructed to walk at their usual pace over a 4-m distance, and the time was recorded using a stopwatch. A gait speed of ≤ 0.8 m/s was considered indicative of impaired physical performance. Alternatively, the SPPB was used, which integrates balance, gait speed, and chair stand tests into a composite score ranging from 0 to 12. A total score of < 9 points was considered to reflect reduced physical performance.

For patients who were unable to ambulate independently, particularly those who were bedridden, physical performance impairment was considered present by default.

### Statistical analyses

All statistical analyses were conducted using SPSS version 26.0 (IBM Corp., Chicago, IL, United States) and R version 4.4.2 (R Foundation for Statistical Computing, Vienna, Austria). A two-tailed *P* < 0.05 was considered statistically significant.

Continuous variables were expressed as mean ± standard deviation (mean ± SD) when normally distributed, and intergroup comparisons were performed using the independent samples *t*-test. Non-normally distributed data were presented as median with interquartile range [M (P25, P75)] and analyzed using the Mann–Whitney U test.

The association between ordinal variables was assessed using Spearman’s rank correlation coefficient (ρ). The reliability of repeated ultrasound measurements was evaluated using intraclass correlation coefficients (ICCs). ICC values for absolute agreement were interpreted as follows: < 0.50, poor; 0.50–0.75, moderate; 0.75–0.90, good; and > 0.90, excellent (36).

Univariate and multivariate logistic regression analyses were performed to identify independent predictors of Chronic hemodialysis complicated with sarcopenia. Prior to logistic regression analysis, all continuous variables were standardized using z-score transformation to minimize the influence of differences in measurement scales. Therefore, the reported odds ratios represent the change in risk associated with a one-standard-deviation increase in each predictor rather than a one-unit increase in the original measurement scale.

Multivariable logistic regression models were constructed using a stepwise selection approach. Candidate variables were first screened by univariable analyses, and only variables meeting the predefined selection criteria were entered into the stepwise multivariable model. Considering the relatively limited sample size, this approach was adopted to construct parsimonious models while reducing the risk of overfitting. Internal validation was performed using bootstrap resampling to assess model stability and calibration. The diagnostic performance of skeletal muscle ultrasound parameters for identifying sarcopenia was evaluated using receiver operating characteristic (ROC) curve analysis. Optimal cutoff values were determined by maximizing the Youden index. Sensitivity, specificity, positive predictive value (PPV), negative predictive value (NPV), and overall accuracy were calculated accordingly.

## Results

### Baseline characteristics

Between January 2025 and April 2025, a total of 126 MHD subjects were enrolled at Lishui People’s Hospital based on the inclusion and exclusion criteria. The mean age of the participants was 61.00 (55.00, 70.00) years, the mean BMI was 22.04 (20.52, 25.07) kg/m^2^, and the mean handgrip strength was 21.55 (17.35, 29.00) kg.

According to the sarcopenia diagnostic criteria, 64 subjects were classified into the no-sarcopenia group and 62 into the sarcopenia group. The mean handgrip strength was 29.00 (23.17, 33.20) kg in the no-sarcopenia group and 17.25 (15.20, 21.23) kg in the sarcopenia group. There were no statistically significant differences between the two groups in terms of gender, BMI, or history of hemodialysis; however, there were statistically significant differences in terms of age, grip strength, and underlying causes of kidney disease.

Serological test results showed that only the difference in albumin levels between the groups was statistically significant, with a mean of 38.450 (35.850, 41.925) g/L in the sarcopenia group and 40.550 (38.075, 43.625) g/L in the non-sarcopenia group. Differences in all other parameters between the groups were not statistically significant, as detailed in Table 1.

**TABLE 1:** Baseline clinical profile of MHD patients.

Variables	No-sarcopenia group (*n* = 64)	Sarcopenia group (*n* = 62)	Statistic	*P*
Gender, *n*(%)			χ^2^ = 1.112	0.292
Female	23 (35.938)	28 (45.161)		
Male	41 (64.062)	34 (54.839)		
Age (year)	59.500 (50.750, 66.250)	65.000 (58.250, 74.000)	*Z* = −3.230	0.001
BMI (kg/m^2^)	22.449 (20.407, 25.025)	21.780 (20.547, 25.150)	*Z* = −0.098	0.922
Handgrip strength (kg)	29.000 (23.175, 33.200)	17.250 (15.200, 21.225)	*Z* = −7.845	<0.001
Hemodialysis duration (year)	5.000 (2.000, 7.000)	3.000 (2.000, 5.000)	*Z* = −1.609	0.108
Primary cause of kidney disease, n(%)			χ^2^ = 14.395	0.006
Hypertensive nephrosclerosis	16 (25.000)	7 (11.290)		
Diabetic nephropathy	10 (15.625)	18 (29.032)		
Polycystic kidney disease	4 (6.250)	3 (4.839)		
Chronic glomerulonephritis	30 (46.875)	19 (30.645)		
Drug-induced kidney disease	4 (6.250)	15 (24.194)		
TC (mmol/L)	4.086 ± 1.038	4.141 ± 1.094	*t* = −0.286	0.775
LDL (mmol/L)	2.092 ± 0.722	2.122 ± 0.763	*t* = −0.232	0.817
BUN (mmol/L)	10.350 (5.840, 20.485)	17.450 (6.592, 22.837)	*Z* = −1.723	0.085
Cr (μmol/L)	582.500 (356.750, 866.250)	680.500 (357.000, 848.250)	*Z* = −0.010	0.992
Alb (g/L)	40.550 (38.075, 43.625)	38.450 (35.850, 41.925)	*Z* = −2.806	0.005
Hb (g/L)	118.500 (109.000, 130.000)	116.500 (106.250, 127.000)	*Z* = −0.644	0.519
PTH (pg/mL)	183.950 (93.225, 325.250)	182.300 (111.000, 334.000)	*Z* = −0.037	0.971
P (mmol/L)	1.630 (1.277, 1.900)	1.550 (1.147, 1.835)	*Z* = −0.910	0.363
UA (μmol/L)	153.500 (97.900, 414.250)	361.900 (115.000, 425.000)	*Z* = −1.364	0.173
HDL (mmol/L)	0.955 (0.835, 1.115)	1.075 (0.830, 1.218)	*Z* = −1.149	0.250

### Repeatability assessment results of ultrasound parameters

We randomly selected 30 patients to assess the reliability of ultrasound findings. By comparing the two sets of measurements for subjects, the ICCs of RF-MT, RF-CSA, RF-SFT, RF-SWV, VI-MT, RA-MT, RA-CSA, RA-SFT, RA-SWV, MG-MT, MG-SFT, PA, MG-ML, MG-SWV, BB-MT, BB-CSA, BB-SFT, and BB-SWV were 0.995, 0.937, 0.987, 0.907, 0.989, 0.981, 0.962, 0.994, 0.894, 0.987, 0.966, 0.872, 0.912, 0.869, 0.985, 0.951, 0.972, and 0.892, respectively. The results indicate that all examination parameters for peripheral skeletal muscles demonstrated excellent reproducibility and high reliability.

### Intergroup differences and correlations in ultrasound parameters

**FIGURE 3:**
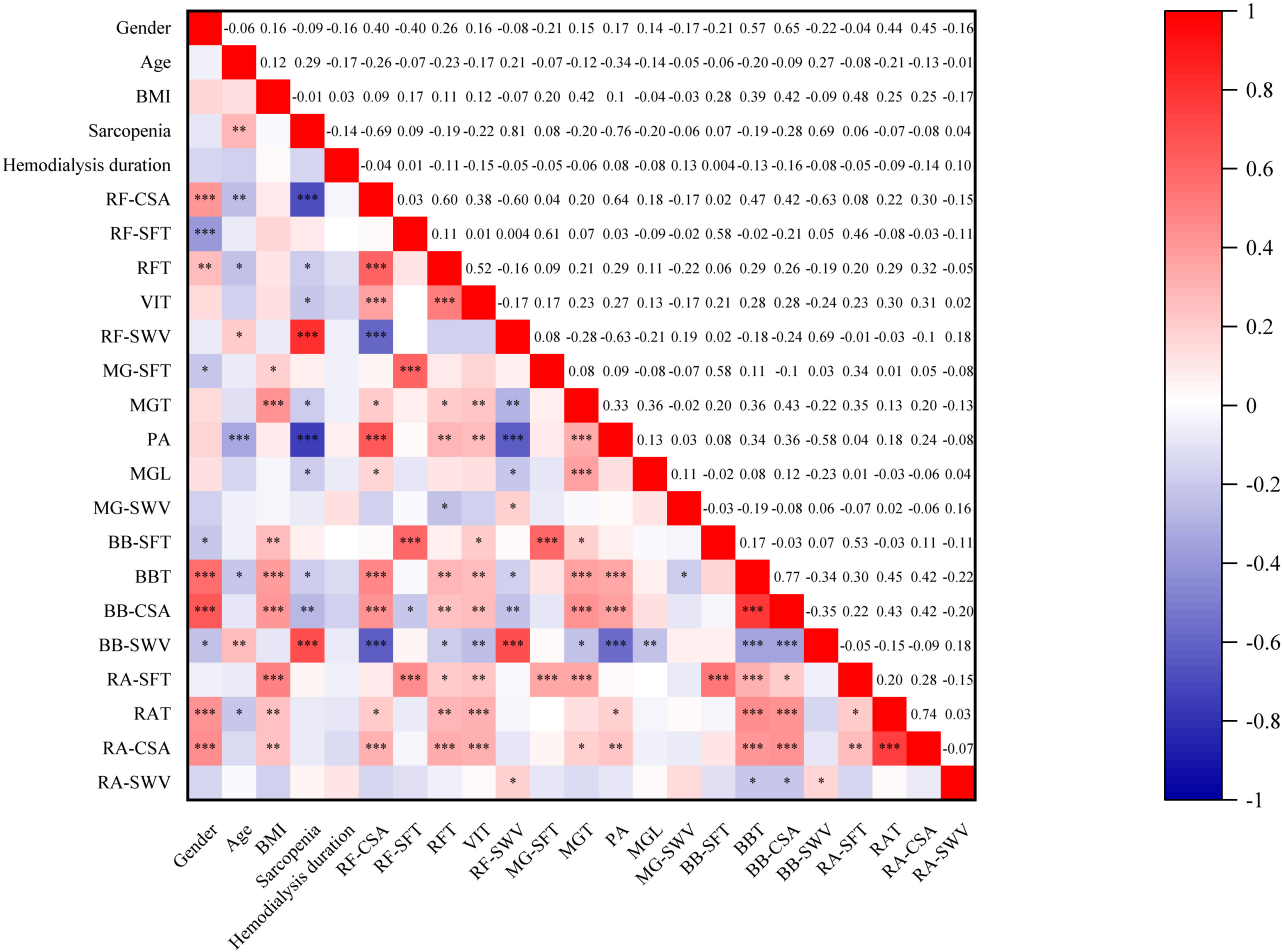
Heatmap of Spearman’s correlation coefficients between parameters. * Indicates *P* < 0.05, ** indicates *P* < 0.01, and *** indicates *P* < 0.005.

The two-dimensional ultrasound parameters of all subjects were analyzed. Intergroup comparisons were performed using the rank-sum test combined with Spearman’s correlation analysis. The results showed that RF-MT (*P* = 0.012, *r* = −0.19), RF-CSA (*P* < 0.001, *r* = −0.69), VI-MT (*P* = 0.009, *r* = −0.22), MG-MT (*P* = 0.027, *r* = −0.20), MG-ML (*P* = 0.014, *r* = −0.20), MG-PA (*P* < 0.001, *r* = −0.76), BB-MT (*P* = 0.031, *r* = −0.19), and BB-CSA (*P* = 0.002, *r* = −0.28) were all significantly associated with sarcopenia in MHD patients, as shown in Table 2 and Figure 3.

**TABLE 2:** Differences analysis of ultrasound parameters in sarcopenia patients.

Variables	No-sarcopenia group (*n* = 64)	Sarcopenia group (*n* = 62)	Statistic	*P*
RF-MT (cm)	1.245 ± 0.284	1.119 ± 0.271	*t* = 2.555	0.012
RF-CSA (cm^2^)	5.560 (4.815, 6.412)	3.365 (2.492, 4.247)	*Z* = −7.752	<0.001
RF-SWV (m/s)	1.820 (1.425, 2.138)	3.125 (2.762, 3.362)	*Z* = −9.094	<0.001
RF-SFT (cm)	0.500 (0.390, 0.658)	0.580 (0.412, 0.730)	*Z* = −0.976	0.329
VI-MT (cm)	1.350 ± 0.383	1.177 ± 0.345	*t* = 2.650	0.009
MG-MT (cm)	1.420 (1.230, 1.600)	1.295 (1.080, 1.520)	*Z* = −2.216	0.027
MG-ML (cm)	4.137 ± 0.532	3.877 ± 0.636	*t* = 2.485	0.014
MG-SWV (m/s)	2.359 ± 0.481	2.332 ± 0.683	*t* = 0.258	0.797
MG-PA (°)	24.000 (22.000, 28.000)	20.200 (18.000, 23.000)	Z = −4.966	<0.001
MG-SFT (cm)	0.260 (0.208, 0.390)	0.290 (0.210, 0.398)	*Z* = −0.872	0.383
BB-MT (cm)	1.880 (1.520, 2.147)	1.675 (1.415, 1.990)	*Z* = −2.155	0.031
BB-CSA (cm^2^)	7.470 (5.612, 9.117)	5.920 (4.772, 7.520)	*Z* = −3.091	0.002
BB-SWV (m/s)	2.835 (2.428, 3.647)	4.610 (4.238, 5.518)	*Z* = −7.681	<0.001
BB-SFT (cm)	0.245 (0.198, 0.340)	0.285 (0.200, 0.380)	*Z* = −0.786	0.432
RA-MT (cm)	0.845 (0.700, 1.020)	0.810 (0.662, 0.970)	*Z* = −0.771	0.441
RA-CSA (cm^2^)	4.075 (3.225, 4.985)	3.910 (2.922, 4.805)	*Z* = −0.837	0.403
RA-SWV (m/s)	2.875 (2.413, 3.303)	2.925 (2.560, 3.280)	*Z* = −0.478	0.632
RA-SFT (cm)	1.305 (1.038, 1.843)	1.485 (1.010, 1.958)	*Z* = −0.637	0.524

For SWE parameters, intergroup analysis using the rank-sum test and Spearman’s correlation indicated that RF-SWV (*P* < 0.001, *r* = 0.81) and BB-SWV (*P* < 0.001, *r* = 0.69) were significantly correlated with sarcopenia, whereas MG-SWV and RA-SWV showed no significant associations (*P* > 0.05). Detailed results are presented in Table 2 and Figure 3.

### Muscle ultrasound determinants for diagnosing sarcopenia in MHD patients

Our multivariable logistic regression analysis of two-dimensional ultrasound and clinical parameters identified gender (1 = male, 0 = female) (*P* = 0.002, OR = 25.206), hemodialysis duration (*P* = 0.005, OR = 0.778), RF-CSA (*P* < 0.001, OR = 0.029), BB-SFT (*P* = 0.004, OR = 2.401), and BB-CSA (*P* = 0.028, OR = 0.391) as independent predictors of sarcopenia in patients undergoing MHD (Table 3).

**TABLE 3:** Univariate and multivariate logistic regression analysis of two-dimensional ultrasound parameters in MHD-associated sarcopenia.

Variables	Univariate regression			Multiple factor regression		
	β	*P*	OR (95%CI)	β	*P*	OR (95%CI)
Gender, n(%)						
Female			1.000 (Reference)			1.000 (Reference)
Male	−0.384	0.292	0.681 (0.333∼1.392)	3.227	0.002	25.206 (3.371∼188.458)
Age (year)	0.053	0.002	1.054 (1.020∼1.090)	0.020	0.568	
Hemodialysis duration (year)	−0.067	0.187	0.935 (0.846∼1.033)	−0.251	0.005	0.778 (0.652∼0.929)
BMI (kg/m^2^)	−0.016	0.927	0.984 (0.693∼1.397)			
Alb (g/L)	−0.050	0.156	0.952 (0.888∼1.019)			
RF-CSA (cm^2^)	−2.294	<0.001	0.101 (0.047∼0.218)	−3.548	<0.001	0.029 (0.008∼0.104)
RF-SFT (cm)	−0.009	0.960	0.991 (0.698∼1.408)			
VI-MT (cm)	−0.498	0.012	0.608 (0.413∼0.895)	0.085	0.839	
MG-PA (°)	−1.011	<0.001	0.364 (0.226∼0.586)	−0.639	0.113	
BB-SFT (cm)	0.141	0.442	1.152 (0.804∼1.650)	0.876	0.004	2.401 (1.329∼4.335)
BB-CSA (cm^2^)	−0.537	0.007	0.584 (0.395∼0.864)	−0.939	0.028	0.391 (0.169∼0.904)
RA-SFT (cm)	0.046	0.799	1.047 (0.737∼1.486)			
RA-CSA (cm^2^)	−0.112	0.532	0.894 (0.628∼1.272)			
MG-SFT (cm)	0.144	0.425	1.155 (0.810∼1.648)			

**FIGURE 4:**
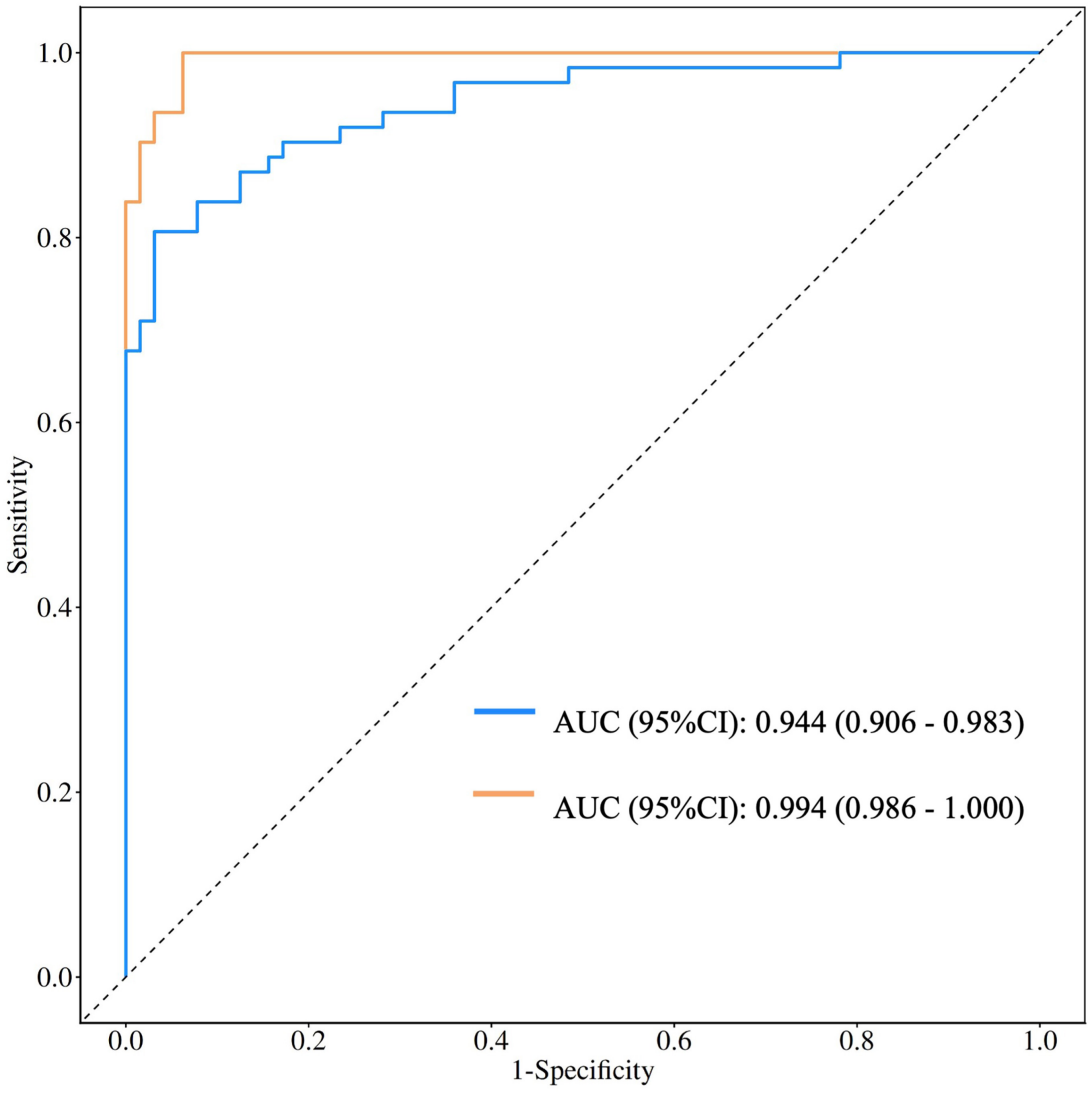
Comparison of AUC curves between models.

The logistic regression model based on two-dimensional ultrasound parameters demonstrated strong discriminative ability, with an AUC of 0.944 (95% CI: 0.906–0.983), as shown in Figure 4. The optimal cutoff value, determined using the Youden index, yielded an accuracy of 0.889 (95% CI: 0.821–0.938), sensitivity of 0.969 (95% CI: 0.926–1.000), specificity of 0.806 (95% CI: 0.708–0.905), PPV of 0.838 (95% CI: 0.754–0.922), and NPV of 0.962 (95% CI: 0.909–1.000) (Table 4).

**TABLE 4:** Comparison of confusion matrices for the two models.

Models	Two-dimensional ultrasound	Combined elasticity ultrasound
AUC (95%CI)	0.944 (0.906–0.983)	0.994 (0.986–1.000)
Cutoff values	0.636	0.276
Accuracy (95%CI)	0.889 (0.821–0.938)	0.968 (0.921–0.991)
Sensitivity (95%CI)	0.969 (0.926–1.000)	1.000 (1.000–1.000)
Specificity (95%CI)	0.806 (0.708–0.905)	0.938 (0.878–0.997)
PPV (95%CI)	0.838 (0.754–0.922)	0.939 (0.882–0.997)
NPV (95%CI)	0.962 (0.909–1.000)	1.000 (1.000–1.000)

We then included additional elasticity ultrasound parameters in our logistic regression analysis and found that hemodialysis duration (*P* = 0.008, OR = 0.536), RF-CSA (*P* = 0.005, OR = 0.036), and RF-SWV (*P* < 0.001, OR = 245.16) were independent predictors of sarcopenia in patients undergoing MHD (Table 5). Interestingly, the inverse association between hemodialysis duration and sarcopenia observed in this study may reflect survivor bias or patient selection effects, as patients with longer dialysis duration who remain clinically stable may represent a relatively healthier subgroup.

**TABLE 5:** Univariate and multivariate logistic regression analysis of two-dimensional and elasticity ultrasound parameters in MHD-associated sarcopenia.

Variables	Univariate regression			Multiple factor regression		
	β	*P*	OR (95%CI)	β	*P*	OR (95%CI)
Gender, n(%)						
Female			1.000 (Reference)			
Male	−0.384	0.292	0.681 (0.333∼1.392)			
Age (year)	0.053	0.002	1.054 (1.020∼1.090)	0.020	0.568	
BMI (kg/m^2^)	−0.004	0.927	0.996 (0.907∼1.094)			
Alb (g/L)	−0.050	0.156	0.952 (0.888∼1.019)			
Hemodialysis duration (year)	−0.067	0.187	0.935 (0.846∼1.033)	−0.624	0.008	0.536 (0.339∼0.848)
RF-MT (cm)	−0.472	0.014	0.624 (0.427∼0.910)	0.491	0.359	
RF-CSA (cm^2^)	−1.507	<0.001	0.222 (0.134∼0.367)	−3.316	0.005	0.036 (0.004∼0.370)
RF-SWV (m/s)	4.839	<0.001	126.282 (18.440∼864.797)	5.502	<0.001	245.160 (12.380∼4854.811)
RF-SFT (cm)	−0.023	0.960	0.977 (0.398∼2.399)			
VI-MT (cm)	−1.332	0.012	0.264 (0.094∼0.743)	0.085	0.839	
MG-SFT (cm)	1.069	0.425	2.913 (0.211∼40.279)			
MG-PA (°)	−0.197	<0.001	0.821 (0.749∼0.901)	−0.639	0.113	
RA-SFT (cm)	0.069	0.799	1.071 (0.630∼1.823)			
RA-CSA (cm^2^)	−0.077	0.532	0.926 (0.729∼1.178)			
BB-SFT (cm)	0.947	0.442	2.578 (0.231∼28.816)			
BB-CSA (cm^2^)	−0.537	0.007	0.584 (0.395∼0.864)	−1.025	0.068	
BB-SWV (m/s)	2.426	<0.001	11.314 (4.957∼25.824)	0.353	0.579	

When elasticity parameters were integrated with two-dimensional ultrasound, the combined model exhibited improved diagnostic performance, achieving an AUC of 0.994 (95% CI: 0.986–1.000), as shown in Figure 4. The corresponding accuracy increased to 0.968 (95% CI: 0.921–0.991), with a sensitivity of 1.000 (95% CI: 1.000–1.000) and specificity of 0.938 (95% CI: 0.878–0.997). Additionally, the PPV and NPV were 0.939 (95% CI: 0.882–0.997) and 1.000 (95% CI: 1.000–1.000). The corresponding optimal cutoff value and diagnostic performance metrics are summarized in Table 4.

The DeLong test demonstrated that the addition of shear wave elastography parameters significantly improved the diagnostic performance of the model compared with the conventional ultrasound model alone (*P* = 0.0074).

Although the combined model demonstrated excellent discriminative performance in this cohort, the findings should be interpreted cautiously owing to the relatively small sample size, the use of stepwise regression, and the lack of external validation may have increased the risk of model overfitting.

### Model robustness and validation

**FIGURE 5:**
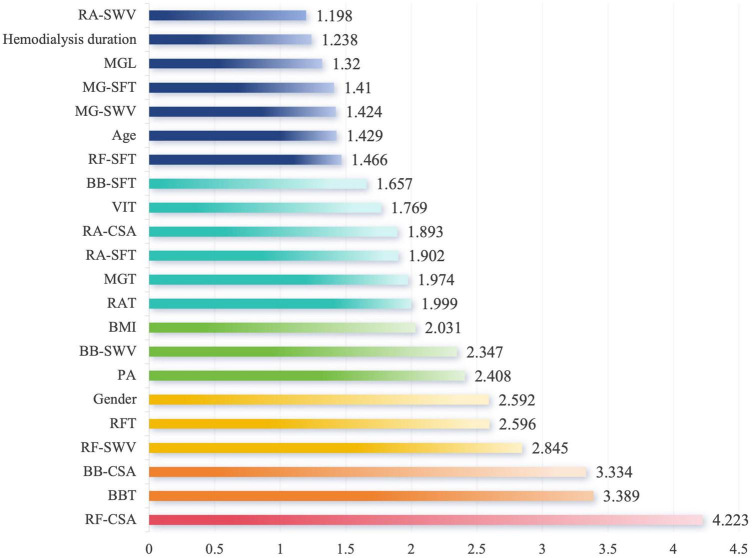
Variance inflation factor (VIF) plots.

To evaluate the stability and reliability of the predictive models, we conducted a series of internal validation procedures, including multicollinearity assessment and goodness-of-fit testing. Specifically, Spearman correlation heatmaps (Figure 3) were used to illustrate the relationships among variables and to identify potential linear associations between predictors. Variance inflation factor (VIF) plots (Figure 5) were further applied to assess multicollinearity, with all variables showing VIF values below 5, indicating no significant collinearity concerns.

**FIGURE 6:**
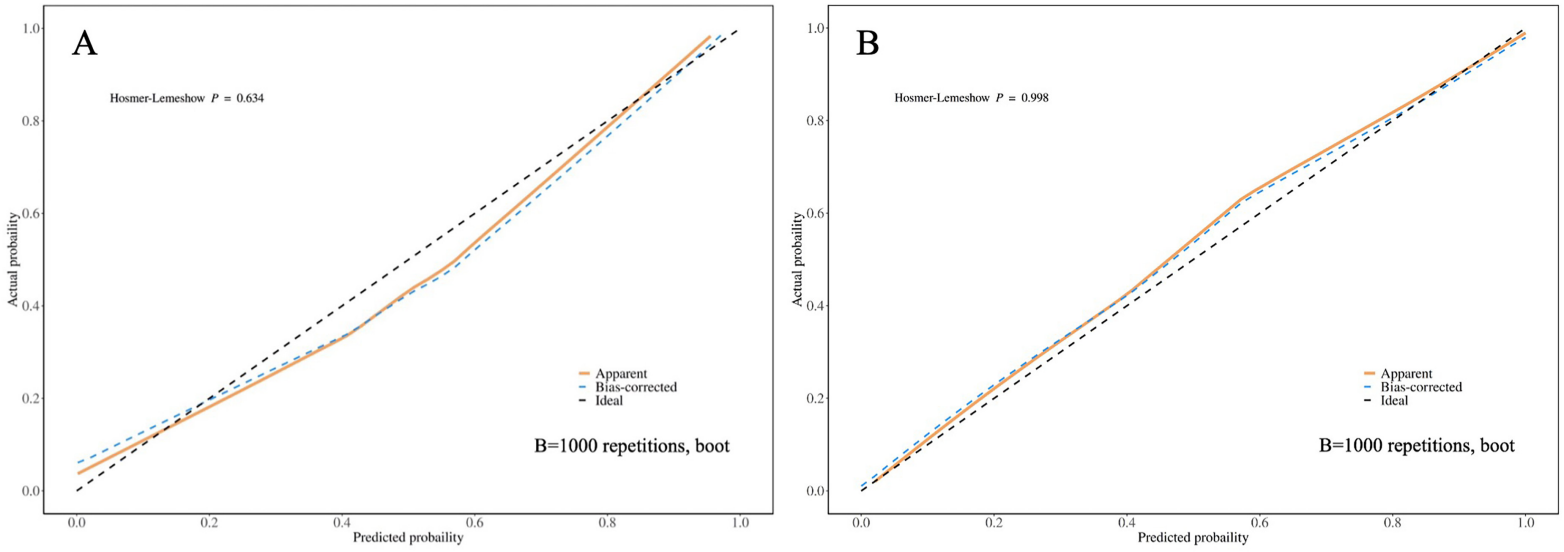
Bootstrap-based Hosmer-Lemeshow test plots for the two-dimensional ultrasound **(A)** and combined elasticity models **(B)**.

Model calibration was examined using a bootstrap-based Hosmer–Lemeshow test (Figures 6A,B). The resulting *p*-values were greater than 0.05 for both the two-dimensional (Figure 6A) model and the combined elasticity ultrasound (Figure 6B) model, suggesting an adequate fit to the observed data.

Overall, these validation results support the robustness of the models, demonstrating low multicollinearity and satisfactory goodness-of-fit in the internal validation process.

## Discussion

This prospective study systematically evaluated the feasibility and diagnostic value of musculoskeletal ultrasound, incorporating both conventional two-dimensional and shear wave elastography parameters, for the assessment of sarcopenia in patients undergoing maintenance hemodialysis. The results demonstrate that ultrasound-derived muscle parameters exhibit excellent reproducibility, that significant structural and mechanical differences exist between sarcopenic and non-sarcopenic patients across multiple muscle groups, and that the integration of SWE substantially enhances diagnostic performance.

A key strength of this study lies in the high reliability of ultrasound measurements, with intraclass correlation coefficients ranging from 0.869 to 0.995. Given the operator-dependent nature of ultrasound, this level of reproducibility is particularly important and supports the feasibility of implementing standardized scanning protocols in clinical practice. These findings indicate that, when performed by trained operators under controlled conditions, musculoskeletal ultrasound can provide stable and repeatable quantitative assessments of skeletal muscle in dialysis patients (37, 38).

In terms of diagnostic relevance, parameters reflecting muscle quantity, particularly rectus femoris cross-sectional area (RF-CSA), showed strong associations with sarcopenia and consistently emerged as independent predictors in multivariable models. This is consistent with previous studies, which have highlighted the quadriceps as a sensitive indicator of systemic muscle loss (39–41). The strong inverse relationship between RF-CSA and sarcopenia underscores its potential role as a core imaging biomarker (42). In addition, several anatomical structures and multi-site parameters, including pennation angle, fascicle length, and biceps brachii measurements, have been found to be associated with sarcopenia, suggesting that muscle atrophy in patients undergoing maintenance hemodialysis is not limited to specific sites but represents a systemic and heterogeneous pathological process (43, 44). This supports the rationale for multi-muscle assessment strategies, as single-site measurements may not fully capture the complexity of muscle alterations in this population.

An important contribution of this study is the demonstration of the added value of SWE in sarcopenia assessment. Among elasticity-related parameters, rectus femoris shear wave velocity (RF-SWV) showed a particularly strong association with sarcopenia and remained an independent predictor after adjustment. The incorporation of SWE markedly improved model performance, with the area under the curve increasing from 0.944 to 0.994. The large odds ratio observed for RF-SWV likely reflects the use of z-score standardization and the strong discriminative ability of this parameter in the current dataset. Nevertheless, the exceptionally high AUC observed in the present study should be interpreted cautiously. Given the relatively small sample size, the use of stepwise regression, and the absence of external validation, the possibility of model overfitting cannot be completely excluded, and independent multicenter validation is required before routine clinical application.

This finding suggests that muscle stiffness provides complementary information beyond traditional morphological parameters. From a pathophysiological perspective, increased shear wave velocity may reflect microstructural alterations such as fibrosis, fatty infiltration, or changes in muscle composition, which are not detectable by thickness or cross-sectional area measurements alone (45–47). These structural changes may increase passive muscle stiffness, impair contractile efficiency, and ultimately contribute to reduced muscle strength and physical performance, the key functional characteristics of sarcopenia. In patients undergoing maintenance hemodialysis, chronic inflammation, metabolic acidosis, uremic toxin accumulation, and reduced physical activity may further promote these pathological alterations, thereby affecting the mechanical properties of skeletal muscle. Therefore, SWV may provide complementary information regarding muscle quality in addition to conventional ultrasound measurements of muscle quantity.

It is noteworthy that not all SWE parameters demonstrated equivalent diagnostic utility. While rectus femoris and biceps brachii shear wave velocities were significantly associated with sarcopenia, elasticity measurements of the gastrocnemius and rectus abdominis did not show significant differences between groups. It is noteworthy that not all SWE parameters demonstrated equivalent diagnostic utility. While rectus femoris and biceps brachii shear wave velocities were significantly associated with sarcopenia, elasticity measurements of the gastrocnemius and rectus abdominis did not show significant differences between groups. This variability may be attributed to anatomical and technical factors, including muscle depth, anisotropy, probe pressure sensitivity, and differences in fiber orientation (48, 49). These findings highlight that the selection of target muscles is critical when applying SWE in clinical settings and suggest that the rectus femoris may be a particularly robust and reliable site for stiffness assessment. In contrast, the gastrocnemius demonstrated lower discriminative ability, which may be related to its greater anatomical variability, pennate architecture, and distinct functional role as an antigravity muscle that remains relatively active during daily standing and walking. These characteristics may reduce the sensitivity of gastrocnemius measurements for detecting sarcopenia-related changes. In addition, shear wave elastography was not performed for the vastus intermedius because its deep anatomical location increases signal attenuation and reduces measurement reliability, highlighting one of the current technical limitations of ultrasound elastography (48, 49).

These findings further suggest that the choice of target muscles may substantially influence the performance of ultrasound-based predictive models. Muscles that are more susceptible to sarcopenia-related structural and mechanical alterations, such as the rectus femoris, are likely to provide greater diagnostic discrimination than muscles that are less consistently affected. Therefore, careful selection of target muscles is an important consideration when developing simplified and clinically applicable ultrasound screening protocols.

From a clinical standpoint, the findings of this study support the use of musculoskeletal ultrasound be integrated into routine dialysis care as an initial bedside screening tool to identify patients who would benefit from comprehensive sarcopenia assessment. Patients identified as high risk based on ultrasound findings could subsequently undergo comprehensive assessment using established diagnostic criteria, including muscle strength and physical performance evaluation. Such a stepwise approach may facilitate early risk stratification while reducing reliance on more costly or less accessible imaging modalities.

Compared with conventional methods such as dual-energy X-ray absorptiometry, bioelectrical impedance analysis, computed tomography, and magnetic resonance imaging, ultrasound offers several advantages, including portability, cost-effectiveness, absence of radiation exposure, and reduced susceptibility to fluid status fluctuations. These features are especially relevant in dialysis populations, in whom fluid overload can compromise the accuracy of other body composition assessment techniques. The high diagnostic accuracy observed in the combined model suggests that ultrasound could serve as a promising first-line screening modality, enabling early identification of high-risk individuals and facilitating timely intervention. Notably, rectus femoris-derived parameters, particularly RF-CSA and RF-SWV, showed the strongest associations with sarcopenia and remained independent predictors in the final models. These findings suggest that a simplified ultrasound protocol focusing on the rectus femoris may represent a practical approach for sarcopenia screening in maintenance hemodialysis patients and warrants further investigation.

Because fluid status may influence both muscle thickness and tissue stiffness measurements in patients undergoing maintenance hemodialysis, all ultrasound examinations were performed after completion of the dialysis session to minimize the effects of fluid overload. However, ultrafiltration and interdialytic weight gain may still influence muscle morphology and elasticity measurements, and post-dialysis assessment can reduce, but not completely eliminate, this source of variability. Future studies comparing ultrasound measurements obtained before and after dialysis are warranted to evaluate inter-session stability and further optimize measurement standardization.

Interestingly, a negative association between hemodialysis duration and sarcopenia was observed in our study. This finding should be interpreted cautiously, as it may reflect survivor bias, selection bias, or the relatively small sample size, whereby patients who survive longer on maintenance hemodialysis and remain clinically stable may represent a healthier subgroup.

This study provides a comprehensive evaluation by incorporating multiple muscle groups and elasticity measurements. However, several limitations should be acknowledged. First, this was a single-center study with a relatively small sample size. In addition, the study population was relatively homogeneous with respect to ethnicity, dietary habits, and dialysis protocol, which may limit the generalizability of our findings to broader and more diverse patient populations and increase the potential for model selection bias associated with stepwise regression. Second, although excellent inter-observer reproducibility was demonstrated, musculoskeletal ultrasound remains operator-dependent, and intra-observer reliability was not assessed. Third, the cross-sectional design precluded the assessment of longitudinal changes and causal relationships, and potential confounding factors, including residual hydration variability related to ultrafiltration and interdialytic weight gain, physical activity, inflammatory burden, and nutritional status, were not comprehensively evaluated. Finally, although DXA was used as the reference method, its accuracy in maintenance hemodialysis patients may be affected by fluid status, and certain deep muscles could not be evaluated using SWE due to technical limitations.

Future studies should validate our findings in larger multicenter cohorts, investigate the prognostic value of ultrasound parameters, and explore the feasibility of simplified rectus femoris-based ultrasound screening protocols.

## Conclusion

In conclusion, this study suggests that musculoskeletal ultrasound may represent a feasible and noninvasive approach for sarcopenia screening in patients undergoing maintenance hemodialysis. The addition of shear wave elastography showed promising incremental value in the present cohort, particularly for rectus femoris assessment. However, given the single-center design and the lack of external validation, these findings should be interpreted with caution. Further validation in larger multicenter cohorts is required before routine clinical implementation.

## Data Availability

The raw data supporting the conclusions of this article will be made available by the authors, without undue reservation.

## References

[1] Ortiz A Yanagita M Yokoi H Torra R. Evolving strategies for early diagnosis, proactive prevention and treatment of CKD. *Nephrol Dial Transplant.* (2026) 41:418–27. 10.1093/ndt/gfaf151 40795360

[2] European Renal Association (ERA) Council Torra R Goumenos DS Arici M Ortiz A Adamczak M et al.. ERA’s ABCDE framework for kidney disease prevention: turning the WHO kidney health resolution into action. *Nephrol Dial Transplant.* (2026) 41:793–801. 10.1093/ndt/gfaf198 41504385 PMC13037474

[3] GBD 2023 Chronic Kidney Disease Collaborators. Global, regional, and national burden of chronic kidney disease in adults, 1990-2023, and its attributable risk factors: a systematic analysis for the Global Burden of Disease Study 2023. *Lancet.* (2025) 406:2461–82. 10.1016/S0140-6736(25)01853-7 41213283

[4] Kidney Disease: Improving Global Outcomes (KDIGO) CKD Work Group. KDIGO 2024 clinical practice guideline for the evaluation and management of chronic kidney disease. *Kidney Int.* (2024) 105:S117–314. 10.1016/j.kint.2023.10.018 38490803

[5] Heitman K Alexander MS Faul C. Skeletal muscle injury in chronic kidney disease-from histologic changes to molecular mechanisms and to novel therapies. *Int J Mol Sci.* (2024) 25:5117. 10.3390/ijms25105117 38791164 PMC11121428

[6] Uchiyama K Wakino S Irie J Miyamoto J Matsui A Tajima T et al.. Contribution of uremic dysbiosis to insulin resistance and sarcopenia. *Nephrol Dial Transplant.* (2020) 35:1501–17. 10.1093/ndt/gfaa076 32535631

[7] Vallianou NG Evangelopoulos AA Christodoulatos GS Tantsi I Mantouvalos N Chatzis D et al.. Unravelling sarcopenia in chronic kidney disease: from pathogenesis to diagnosis and therapeutics. *Diagnostics (Basel).* (2026) 16:1063. 10.3390/diagnostics16071063 41975774 PMC13073154

[8] Bakinowska E Olejnik-Wojciechowska J Kiełbowski K Skoryk A Pawlik A. Pathogenesis of sarcopenia in chronic kidney disease-the role of inflammation, metabolic dysregulation, gut dysbiosis, and microRNA. *Int J Mol Sci.* (2024) 25:8474. 10.3390/ijms25158474 39126043 PMC11313360

[9] Ribeiro HS Neri SGR Oliveira JS Bennett PN Viana JL Lima RM. Association between sarcopenia and clinical outcomes in chronic kidney disease patients: a systematic review and meta-analysis. *Clin Nutr.* (2022) 41:1131–40. 10.1016/j.clnu.2022.03.025 35430544

[10] Duarte MP Almeida LS Neri SGR Oliveira JS Wilkinson TJ Ribeiro HS et al.. Prevalence of sarcopenia in patients with chronic kidney disease: a global systematic review and meta-analysis. *J Cachexia Sarcopenia Muscle.* (2024) 15:501–12. 10.1002/jcsm.13425 38263952 PMC10995263

[11] Shu X Lin T Wang H Zhao Y Jiang T Peng X et al.. Diagnosis, prevalence, and mortality of sarcopenia in dialysis patients: a systematic review and meta-analysis. *J Cachexia Sarcopenia Muscle.* (2022) 13:145–58. 10.1002/jcsm.12890 34989172 PMC8818609

[12] Cruz-Jentoft AJ Sayer AA. Sarcopenia. *Lancet.* (2019) 393:2636–46. 10.1016/S0140-6736(19)31138-9 31171417

[13] Stockings J Heaney S Chu G Choi P Fernandez R. Prevalence and risk factors of sarcopenia in people receiving dialysis: a systematic review and meta-analysis. *Semin Dial.* (2025) 38:237–49. 10.1111/sdi.70000 40625163 PMC12378093

[14] Wathanavasin W Banjongjit A Avihingsanon Y Praditpornsilpa K Tungsanga K Eiam-Ong S et al.. Prevalence of sarcopenia and its impact on cardiovascular events and mortality among dialysis patients: a systematic review and meta-analysis. *Nutrients.* (2022) 14:4077. 10.3390/nu14194077 36235729 PMC9572026

[15] Sabatino A Guerra A Baiocchi de Carvalho KM Cuppari L Carrero JJ Stenvinkel P et al.. Sarcopenia and its individual traits independently predict mortality in patients on dialysis: a systematic review and meta-analysis. *J Cachexia Sarcopenia Muscle.* (2025) 16:e70089. 10.1002/jcsm.70089 41069070 PMC12510902

[16] Sato T Kohzuki M. Rehabilitation for cardiorenal multimorbidity: epidemiology, functional phenotypes, and effects on physical function, renal trajectory, and prognosis. *J Clin Med.* (2026) 15:2504. 10.3390/jcm15072504 41976805 PMC13073499

[17] Phillips T Harris S Aiyegbusi OL Benavente M Cockwell P Kalra PA et al.. Potentially modifiable factors associated with longitudinal health-related quality of life in the National Unified Renal Translational Research Enterprise CKD (NURTuRE-CKD) cohort. *Am J Nephrol.* (2026). 10.1159/000551177 41961751

[18] Massini G Caldiroli L Molinari P Carminati FMI Castellano G Vettoretti S. Nutritional strategies to prevent muscle loss and sarcopenia in chronic kidney disease: what do we currently know? *Nutrients.* (2023) 15:3107. 10.3390/nu15143107 37513525 PMC10384728

[19] Cruz-Jentoft AJ Bahat G Bauer J Boirie Y Bruyère O Cederholm T et al.. Sarcopenia: revised European consensus on definition and diagnosis. *Age Ageing.* (2019) 48:16–31. 10.1093/ageing/afy169 30312372 PMC6322506

[20] Chen LK Woo J Assantachai P Auyeung TW Chou MY Iijima K et al.. Asian Working Group for Sarcopenia: 2019 consensus update on sarcopenia diagnosis and treatment. *J Am Med Dir Assoc.* (2020) 21:300–7.e2. 10.1016/j.jamda.2019.12.012 32033882

[21] Han A Bokshan SL Marcaccio SE DePasse JM Daniels AH. Diagnostic criteria and clinical outcomes in sarcopenia research: a literature review. *J Clin Med.* (2018) 7:70. 10.3390/jcm7040070 29642478 PMC5920444

[22] Stuck AK Basile G Freystaetter G de Godoi Rezende Costa Molino C Lang W Bischoff-Ferrari HA. Predictive validity of current sarcopenia definitions (EWGSOP2, SDOC, and AWGS2) for clinical outcomes: a scoping review. *J Cachexia Sarcopenia Muscle.* (2023) 14:71–83. 10.1002/jcsm.13161 36564353 PMC9891988

[23] Staempfli JS Kistler-Fischbacher M Gewiess J Bastian JD Eggimann AK. The validity of muscle ultrasound in the diagnostic workup of sarcopenia among older adults: a scoping review. *Clin Interv Aging.* (2024) 19:993–1003. 10.2147/CIA.S463917 38831963 PMC11146336

[24] Li S Xu K Wang A Qin C Hua N Ling X et al.. Determination of ultrasound reference values for diagnosing low muscle mass in older Chinese adults. *J Cachexia Sarcopenia Muscle.* (2025) 16:e70155. 10.1002/jcsm.70155 41362106 PMC12686591

[25] Wang JC Wu WT Chang KV Chen LR Chi SY Kara M et al.. Ultrasound imaging for the diagnosis and evaluation of sarcopenia: an umbrella review. *Life (Basel).* (2021) 12:9. 10.3390/life12010009 35054402 PMC8781401

[26] Ticinesi A Meschi T Maggio M Narici MV. Application of ultrasound for muscle assessment in sarcopenia: the challenge of implementing protocols for clinical practice. *Eur Geriatr Med.* (2019) 10:155–6. 10.1007/s41999-018-0126-3 32720272

[27] Fu H Wang L Zhang W Lu J Yang M. Diagnostic test accuracy of ultrasound for sarcopenia diagnosis: a systematic review and meta-analysis. *J Cachexia Sarcopenia Muscle.* (2023) 14:57–70. 10.1002/jcsm.13149 36513380 PMC9891970

[28] Fu H Luo S Zhuo Y Lian R Chen X Jiang W et al.. Enhanced sarcopenia detection in nursing home residents using ultrasound radiomics and machine learning. *J Am Med Dir Assoc.* (2025) 26:105830. 10.1016/j.jamda.2025.105830 40882952

[29] Sabatino A Sola KH Brismar TB Lindholm B Stenvinkel P Avesani CM. Making the invisible visible: imaging techniques for assessing muscle mass and muscle quality in chronic kidney disease. *Clin Kidney J.* (2024) 17:sfae028. 10.1093/ckj/sfae028 38444750 PMC10913944

[30] Shou J Ouyang C Lin J Huang J Yang H He Y. Value of conventional ultrasound combined with shear wave elastography for sarcopenia in patients with cardiovascular disease. *Clin Interv Aging.* (2025) 20:2627–39. 10.2147/CIA.S553232 41459406 PMC12738767

[31] An L Shi J Pan Y Ding Y Gao W Ren L et al.. The role of shear wave elastography in diagnosing sarcopenia in patients with type 2 diabetes. *J Endocrinol Invest.* (2025) 48:2177–85. 10.1007/s40618-025-02623-3 40504330

[32] Zhang C Kang L. Ultrasound elastography for the assessment of sarcopenia. *J Clin Med.* (2026) 15:2566. 10.3390/jcm15072566 41976863 PMC13073094

[33] Dong Z Chen J Ge J Zhao M Zhao Y Zheng S et al.. Respiratory and peripheral muscle ultrasound, including shear-wave elastography, for sarcopenia screening in COPD. *Front Nutr.* (2025) 12:1687103. 10.3389/fnut.2025.1687103 41425629 PMC12715613

[34] Chen ZT Jin FS Guo LH Li XL Wang Q Zhao H et al.. Value of conventional ultrasound and shear wave elastography in the assessment of muscle mass and function in elderly people with type 2 diabetes. *Eur Radiol.* (2023) 33:4007–15. 10.1007/s00330-022-09382-2 36648552

[35] Dag N Berktas HB Uslu AG Burulday V. Sonoelastographic evaluation of diaphragmatic thickness and stiffness in dialysis patients. *BMC Med Imaging.* (2025) 25:380. 10.1186/s12880-025-01932-6 41013361 PMC12465852

[36] Ten Hove D Jorgensen TD van der Ark LA. Updated guidelines on selecting an intraclass correlation coefficient for interrater reliability, with applications to incomplete observational designs. *Psychol Methods.* (2024) 29:967–79. 10.1037/met0000516 36048052

[37] Warneke K Siegel SD Drabow J Lohmann LH Jochum D Freitas SR et al.. Examiner experience moderates reliability of human lower extremity muscle ultrasound measurement - a double blinded measurement error study. *Ultrasound J.* (2025) 17:20. 10.1186/s13089-025-00424-6 40138073 PMC11947354

[38] Van den Broeck J Héréus S Cattrysse E Raeymaekers H De Maeseneer M Scafoglieri A. Reliability of muscle quantity and quality measured with extended-field-of-view ultrasound at nine body sites. *Ultrasound Med Biol.* (2023) 49:1544–9. 10.1016/j.ultrasmedbio.2023.02.018 37002153

[39] Sabatino A Kooman J Avesani CM Gregorini M Bianchi S Regolisti G et al.. Sarcopenia diagnosed by ultrasound-assessed quadriceps muscle thickness and handgrip strength predicts mortality in patients on hemodialysis. *J Nephrol.* (2024) 37:993–1003. 10.1007/s40620-023-01867-7 38263531

[40] Yang Q Zhang C Zhang Z Su B. Muscle ultrasound to diagnose sarcopenia in chronic kidney disease: a systematic review and Bayesian bivariate meta-analysis. *BMC Nephrol.* (2024) 25:12. 10.1186/s12882-023-03445-2 38178026 PMC10768384

[41] Puthucheary ZA Rawal J McPhail M Connolly B Ratnayake G Chan P et al.. Acute skeletal muscle wasting in critical illness. *JAMA.* (2013) 310:1591–600. 10.1001/jama.2013.278481 24108501

[42] Wilkinson TJ Gore EF Vadaszy N Nixon DGD Watson EL Smith AC. Utility of ultrasound as a valid and accurate diagnostic tool for sarcopenia: sex-specific cutoff values in chronic kidney disease. *J Ultrasound Med.* (2021) 40:457–67. 10.1002/jum.15421 32780522

[43] Dong Z Zhao M Sun X Yan X Zheng S Ye J et al.. Prognostic assessment of the COPD patient: it is time to consider respiratory muscle ultrasound. *BMC Pulm Med.* (2026) 26:91. 10.1186/s12890-026-04118-0 41612249 PMC12924214

[44] Güner M Girgin S Ceylan S Özcan B Öztürk Y Okyar Baş A et al.. The role of muscle ultrasonography to diagnose malnutrition and sarcopenia in maintenance hemodialysis. *J Ren Nutr.* (2024) 34:330–6. 10.1053/j.jrn.2023.12.001 38128851

[45] Remuss C Krone JO Giger AW Jepsen DB Andersen-Ranberg K Brockhattingen KK. Shear-wave elastography for muscle assessment in geriatric outpatients at increased risk of falling. *Eur Geriatr Med.* (2026) 17:31–41. 10.1007/s41999-025-01333-6 41129049 PMC12946335

[46] Janczyk EM Champigny N Michel E Raffaelli C Annweiler C Zory R et al.. Sonoelastography to assess muscular stiffness among older adults and its use for the diagnosis of sarcopenia: a systematic review. *Ultraschall Med.* (2021) 42:634–42. 10.1055/a-1293-8057 33187010

[47] Okyar Baş A Baş H Ceylan S Güner Oytun M Koca M Hafızoğlu M et al.. Changes in muscle quality identified by shear-wave elastography and association with sarcopenia. *JPEN J Parenter Enteral Nutr.* (2023) 47:253–64. 10.1002/jpen.2457 36227071

[48] Dong Z Zhao M Shi M Ge J Cao N Chen J et al.. Acoustic guide pad: a simple innovation that dramatically reduces the risk of misdiagnosis in respiratory muscle ultrasound examinations. *Eur J Radiol.* (2026) 199:112767. 10.1016/j.ejrad.2026.112767 41797010

[49] Kim SG Park B Hwang K Jeong WK. Reliability of ultrasound elastography according to experience level and anatomic location. *Clin Orthop Surg.* (2025) 17:166–73. 10.4055/cios24190 39912077 PMC11791484

